# Supplementary material for: DNA metabarcoding of zooplankton communities: species diversity and seasonal variation revealed by 18S rRNA and COI
Source: PeerJ. 2021 Mar 19;9:e11057. doi: 10.7717/peerj.11057 (PMC7983862; doi:10.7717/peerj.11057)
Supplement: Supplemental Information 1 [file peerj-09-11057-s001.docx]

**Table S1** Amplification primers for 18S rRNA and COI.

|  | Primer | Sequence |
| --- | --- | --- |
| 18S | fw | ATTAGGGTTCGATTCCGGAGAGG |
|  | rv | CTGGAATTACCGCGGSTGCTG |
| COI | mlCOIintF | GGWACWGGWTGAACWGTWTAYCCYCC |
|  | jgHCO2198 | TAIACYTCIGGRTGICCRAARAAYCA |
