# Supplementary material for: DNA metabarcoding of zooplankton communities: species diversity and seasonal variation revealed by 18S rRNA and COI
Source: PeerJ. 2021 Mar 19;9:e11057. doi: 10.7717/peerj.11057 (PMC7983862; doi:10.7717/peerj.11057)
Supplement: Supplemental Information 2 [file peerj-09-11057-s002.docx]

**Table S2** The sequencing depth of the two molecular markers.

| **Samples** | **18S** | **COI** |
| --- | --- | --- |
| SUM_1 | 37811 | 24845 |
| SUM_2 | 73434 | 24217 |
| SUM_3 | 68151 | 25145 |
| SUM_4 | 58993 | 20778 |
| SUM_5 | 116411 | 24693 |
| SUM_6 | 114521 | 24451 |
| SUM_7 | 68227 | 19673 |
| SUM_8 | 51802 | 25752 |
| SUM_9 | 75828 | 24747 |
| AUT_1 | 87367 | 31288 |
| AUT_2 | 66996 | 42039 |
| AUT_3 | 81311 | 37210 |
| AUT_4 | 76777 | 28069 |
| AUT_5 | 106031 | 40093 |
| AUT_6 | 65075 | 8730 |
| AUT_7 | 70786 | 14794 |
| AUT_8 | 80422 | 19066 |
| AUT_9 | 78844 | 16739 |
| WIN_1 | 49430 | 18384 |
| WIN_2 | 40987 | 68313 |
| WIN_3 | 57368 | 11795 |
| WIN_4 | 74053 | 17636 |
| WIN_5 | 63424 | 16667 |
| WIN_6 | 36478 | 12109 |
| WIN_7 | 11385 | 21177 |
| WIN_8 | 12890 | 22712 |
| WIN_9 | 20949 | 43639 |
| SPR_1 | 43829 | 46715 |
| SPR_2 | 41172 | 11223 |
| SPR_3 | 45730 | 22832 |
| SPR_4 | 24196 | 41870 |
| SPR_5 | 17819 | 38505 |
| SPR_6 | 5510 | 37702 |
| SPR_7 | 37005 | 7057 |
| SPR_8 | 107898 | 8392 |
| SPR_9 | 116407 | 11080 |
