# Supplementary material for: DNA metabarcoding of zooplankton communities: species diversity and seasonal variation revealed by 18S rRNA and COI
Source: PeerJ. 2021 Mar 19;9:e11057. doi: 10.7717/peerj.11057 (PMC7983862; doi:10.7717/peerj.11057)
Supplement: Supplemental Information 4 [file peerj-09-11057-s004.docx]

**Table S4** Analysis of similarities (ANOSIM) showed seasonal variation in zooplankton community based on Jaccard and Bray-curtis distance.

| **Samples** | **18S** | | | |  | **COI** | | | |
| --- | --- | --- | --- | --- | --- | --- | --- | --- | --- |
|  | **Jaccard distance** | | **Bray-curtis distance** | |  | **Jaccard distance** | | **Bray-curtis distance** | |
|  | R | *p*-value | R | *p*-value |  | R | *p*-value | R | *p*-value |
| SUM vs. AUT | 0.7960 | 0.0001 | 0.7517 | 0.0001 |  | 0.9705 | 0.0001 | 0.8344 | 0.0001 |
| SUM vs. WIN | 1.0000 | 0.0002 | 0.8570 | 0.0003 |  | 1.0000 | 0.0001 | 1.0000 | 0.0002 |
| SUM vs. SPR | 0.8750 | 0.0001 | 0.7771 | 0.0002 |  | 0.9949 | 0.0002 | 0.9462 | 0.0002 |
| AUT vs. WIN | 0.9798 | 0.0002 | 0.8186 | 0.0001 |  | 1.0000 | 0.0001 | 0.9887 | 0.0002 |
| AUT vs. SPR | 0.8712 | 0.0003 | 0.4331 | 0.0002 |  | 0.9863 | 0.0002 | 0.7366 | 0.0001 |
| WIN vs. SPR | 0.5161 | 0.0002 | 0.0988 | 0.1211 |  | 0.7584 | 0.0002 | 0.4462 | 0.0003 |
| Among four seasons | 0.8393 | 0.0001 | 0.5976 | 0.0001 |  | 0.9489 | 0.0001 | 0.8191 | 0.0001 |
