# Supplementary material for: DNA metabarcoding of zooplankton communities: species diversity and seasonal variation revealed by 18S rRNA and COI
Source: PeerJ. 2021 Mar 19;9:e11057. doi: 10.7717/peerj.11057 (PMC7983862; doi:10.7717/peerj.11057)
Supplement: Supplemental Information 5 [file peerj-09-11057-s005.docx]

**Table S5** Adonis analysis shows the contribution and significance of each water environmental factor to the seasonal changes in the relative abundance of zooplankton. *, p < 0.05; **, p < 0.01; ***, p < 0.001.

| Factor | 18S | | |  | COI | | |
| --- | --- | --- | --- | --- | --- | --- | --- |
|  | F | R2 | Pr(>F) |  | F | R2 | Pr(>F) |
| WT | 8.3902 | 0.1979 | 0.001 *** |  | 11.474 | 0.2523 | 0.001 *** |
| pH | 5.4109 | 0.1373 | 0.002 ** |  | 4.1409 | 0.1086 | 0.002 ** |
| COD | 4.6561 | 0.1205 | 0.004 ** |  | 2.7062 | 0.0737 | 0.027 * |
| TP | 3.6491 | 0.0969 | 0.009 ** |  | 4.5738 | 0.1186 | 0.002 ** |
| NH_4_-N | 3.9266 | 0.1035 | 0.008 ** |  | 6.0548 | 0.1517 | 0.002 ** |
| DO | 0.5711 | 0.0165 | 0.666 |  | 1.1911 | 0.0339 | 0.285 |
| TN | 6.1636 | 0.1535 | 0.001 *** |  | 6.2273 | 0.1548 | 0.001 *** |
