# Supplementary figures and images for: DNA metabarcoding of zooplankton communities: species diversity and seasonal variation revealed by 18S rRNA and COI
Source: PeerJ. 2021 Mar 19;9:e11057. doi: 10.7717/peerj.11057 (PMC7983862; doi:10.7717/peerj.11057)

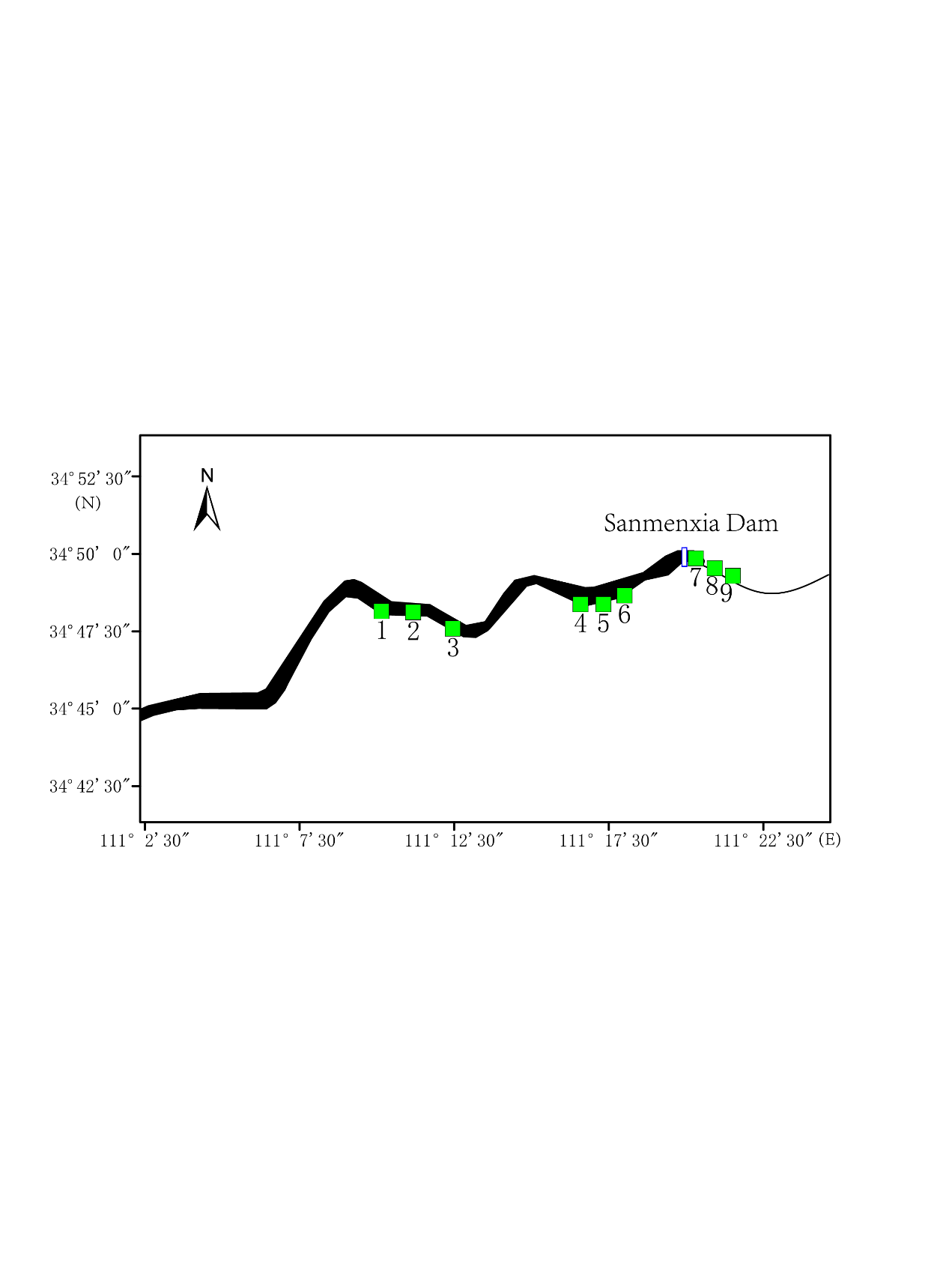


Fig. S1 Sampling sites for zooplankton and water samples in Sanmenxia Reservoir.

Supplement: Supplemental Information 6 [file peerj-09-11057-s006.docx]

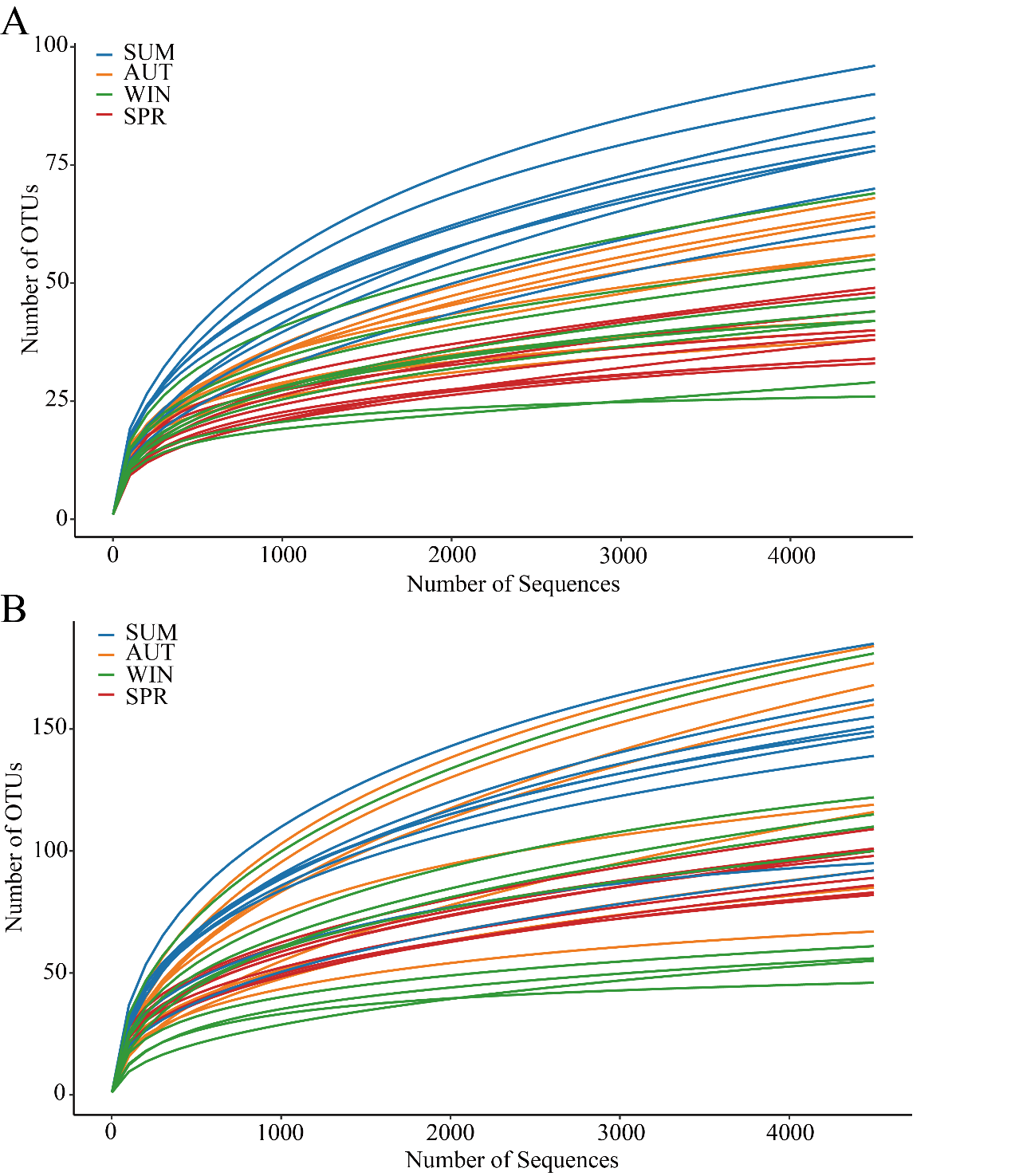


Fig. S2 Rarefaction curve for each sample based on 18S rRNA (A) and COI gene (B).

Supplement: Supplemental Information 7 [file peerj-09-11057-s007.docx]

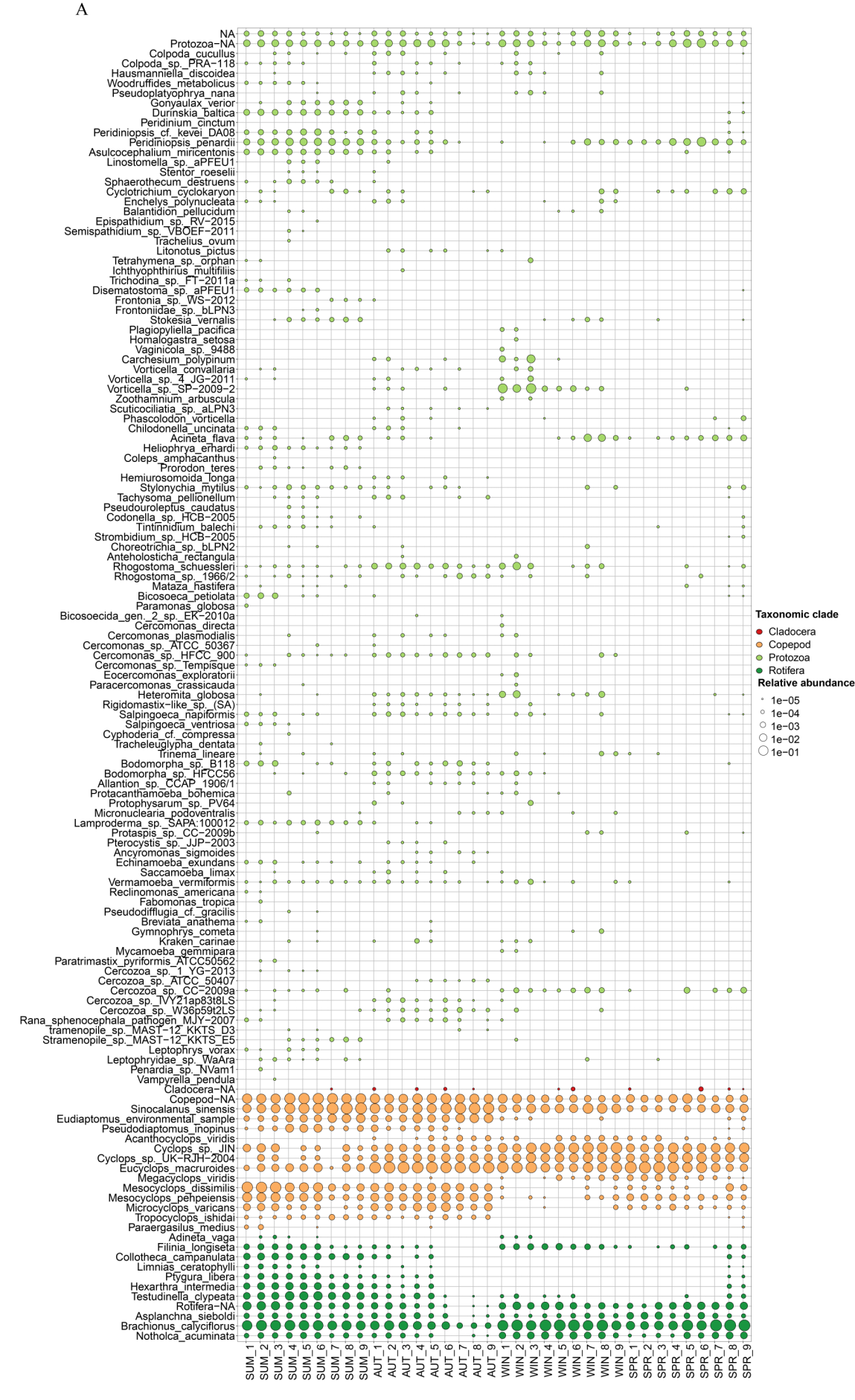


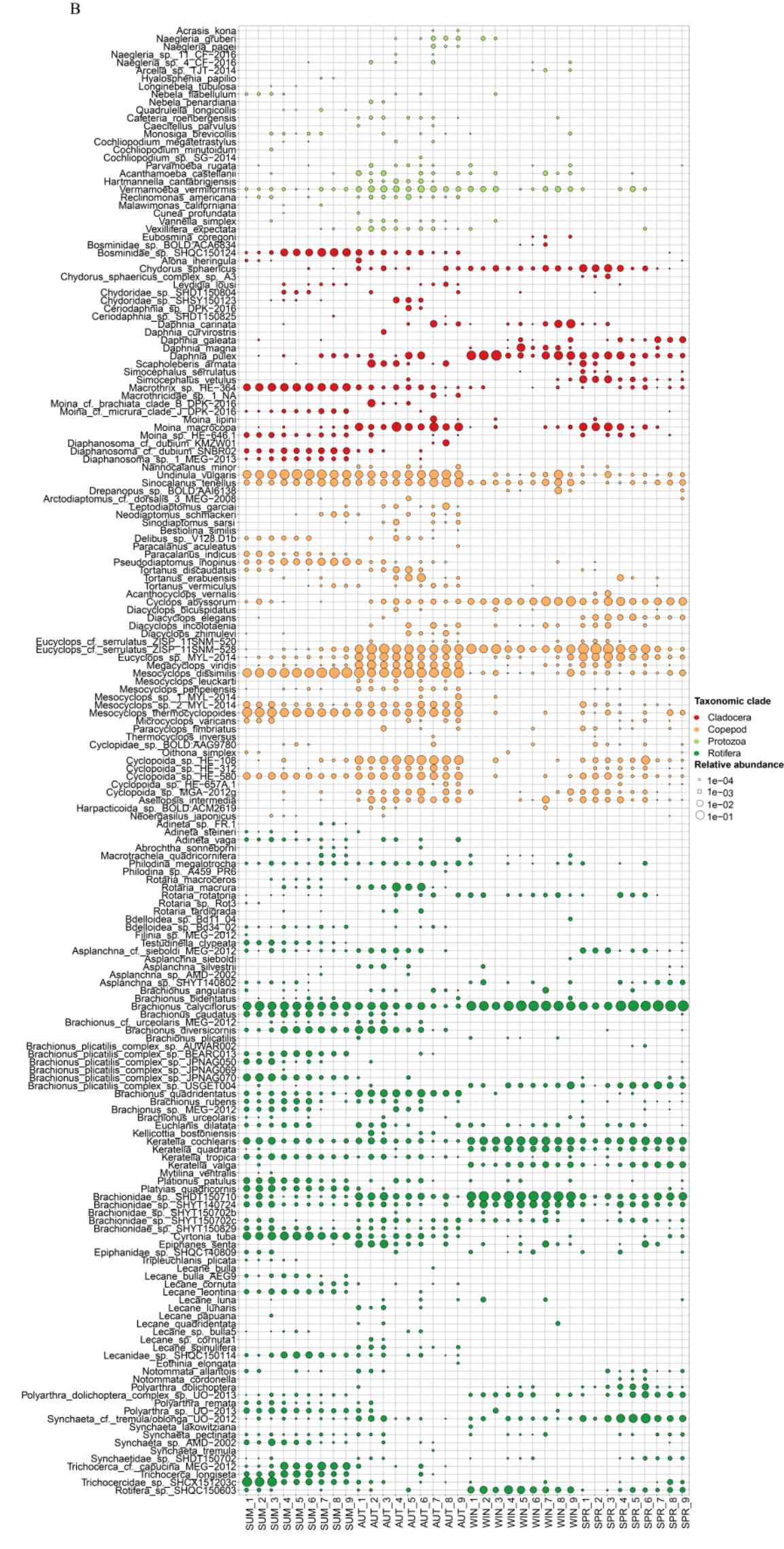


Fig. S3 The seasonal distribution of zooplankton species is sample-based. (A) 18S rRNA; (B) COI.

Supplement: Supplemental Information 8 [file peerj-09-11057-s008.doc]
